# Unique Axon-to-Soma Signaling Pathways Mediate Dendritic Spine Loss and Hyper-Excitability Post-axotomy

**DOI:** 10.3389/fncel.2019.00431

**Published:** 2019-09-24

**Authors:** Tharkika Nagendran, Anne Marion Taylor

**Affiliations:** ^1^UNC/NC State Joint Department of Biomedical Engineering, The University of North Carolina at Chapel Hill, Chapel Hill, NC, United States; ^2^UNC Neuroscience Center, The University of North Carolina at Chapel Hill, Chapel Hill, NC, United States; ^3^Xona Microfluidics, LLC, Research Triangle Park, NC, United States

**Keywords:** retrograde signaling, axon-to-soma, injury model, axotomy, dendritic spine loss, hyper-excitability

## Abstract

Axon damage may cause axon regeneration, retrograde synapse loss, and hyper-excitability, all of which affect recovery following acquired brain injury. While axon regeneration is studied extensively, less is known about signaling mediating retrograde synapse loss and hyper-excitability, especially in long projection pyramidal neurons. To investigate intrinsic injury signaling within neurons, we used an *in vitro* microfluidic platform that models dendritic spine loss and delayed hyper-excitability following remote axon injury. Our data show that sodium influx and reversal of sodium calcium exchangers (NCXs) at the site of axotomy, mediate dendritic spine loss following axotomy. In contrast, sodium influx and NCX reversal alone are insufficient to cause retrograde hyper-excitability. We found that calcium release from axonal ER is critical for the induction of hyper-excitability and inhibition loss. These data suggest that synapse loss and hyper-excitability are uncoupled responses following axon injury. Further, axonal ER may play a critical and underappreciated role in mediating retrograde hyper-excitability within the CNS.

## Introduction

Acute neural injuries (e.g., stroke, traumatic brain injury, and spinal cord injury) cause profound axon damage. Axon damage triggers an intra-cellular signaling cascade to effect neuronal injury responses, including axon regeneration, retrograde synapse loss, and hyper-excitability. These downstream responses are critical for recovery following injury. Yet, the intrinsic neuronal signaling mechanisms mediating retrograde synapse loss and hyper-excitability, in particular, remain poorly understood.

Axon injury induces differential gene expression and transcription within the soma, requiring long range signaling from the site of injury to the nucleus (Rishal and Fainzilber, 2014; Nagendran et al., 2017). Breach of the axonal membrane following axon injury causes an influx of ions, including calcium and sodium, into the intra-axonal space. The increase in sodium ions through voltage-gated sodium channels causes reversal of sodium-calcium exchangers (NCXs) located on the plasma membrane, mitochondria and ER, thus enhancing local intra-axonal calcium levels (Persson et al., 2013). Calcium release from smooth ER within the axon may also potentiate axon-to-soma signaling (Cho et al., 2013; Sun et al., 2014). Axon damage of mouse peripheral sensory neurons was reduced with blockade of both sodium channels and the reverse mode of NCX (Persson et al., 2013), supporting the critical role of NCXs in retrograde injury signaling. Whether local sodium influx and reversal of NCX during axon damage are needed to transmit signals to the soma to cause dendritic spine loss and hyper-excitability remains unknown.

Hippocampal cultures grown within a compartmentalized microfluidic platform provide an injury model system to investigate intrinsic neuronal injury response. These devices guide axonal growth of pyramidal cells, through a microgroove-embedded barrier region of almost 1 mm into an isolated axonal compartment. Because of this barrier region, axons can be injured precisely without mechanically disrupting somatodendritic regions and soluble microenvironments can be established for experimental purposes. Axotomy performed within compartmentalized platforms produced several characteristic injury responses well described *in vivo*, including rapid expression of the immediate early gene *c-fos* (Taylor et al., 2005) and reduced expression of netrin-1 one day following axon damage. Evidence of ER changes within the soma, called chromatolysis, occurs in axotomized neurons *in vitro* (McIlwain and Hoke, 2005; Nagendran et al., 2017). Axotomized neurons within microfluidic chambers also showed that dendritic spine loss (Gao et al., 2011; Ghosh et al., 2012; Nagendran et al., 2017) and hyper-excitability (Frost et al., 2015; Nagendran et al., 2017) occur, providing an experimentally tractable model to study initiation and progression of these neuronal injury responses. Axotomy within these platforms produced a delayed enhancement in neuronal hyper-excitability, 1 day following dendritic spine loss. The extent to which both dendritic spine loss and hyper-excitability use the same retrograde signaling mechanisms is unclear.

## Results

### Sodium Influx and Reversal of Sodium Calcium Exchangers Induce Retrograde Spine Loss

**FIGURE 1:**
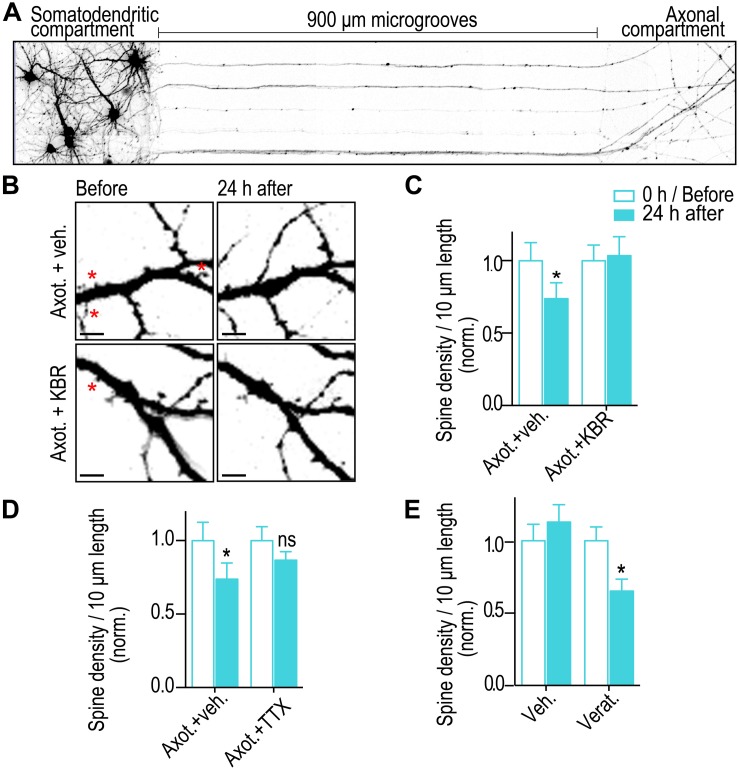
Blocking sodium channels and the reverse mode of NCX at the site of injury prevents axotomy-induced spine loss. **(A)** Inverted gray scale images of 15 DIV rat hippocampal neurons cultured within microfluidic devices that were retrogradely labeled using G-deleted mCherry rabies virus added to axonal compartment. **(B)** Representative images of dendrites before and 24 h after axotomy treated with either vehicle or reversible NCX blocker (KB-R7943; 10 μM). Asterisks indicate eliminated spines. Scale bars, 5 μm. **(C,D)** Quantification of dendritic spine density before and 24 h after axotomy with application of either vehicle or KBR or TTX (1 μM) that was applied only to the axonal compartment for 1 h during injury. **(E)** Quantification of spine density before and 24 h after treatment of axonal compartment with either vehicle or sodium channel activator (veratridine, 10 μM) for 10 min in the absence of injury. *n* = 15 dendrites for each condition over 2 independent experiments. Paired two-tailed *t*-test, ^∗^*p* ≤ 0.05. Error bars, s.e.m.

To selectively injure axons of hippocampal neurons >900 μm from somata, we used compartmentalized microfluidic devices (Figure 1A). Primarily axons of pyramidal neurons are guided via microgrooves into a separated axon compartment where they are injured and various pharmacological treatments can be restricted to isolated axons. We previously found that blocking local activity at the site of injury using this method prevented dendritic spine loss 24 h post-axotomy (Nagendran et al., 2017), suggesting calcium and sodium influx at the site of injury are key mediators of this neuronal injury response. Reversal of NCX at the site of injury may play a key role, causing a massive local influx of calcium. To determine whether local NCX activation is required to trigger retrograde synapse loss, we performed axotomy within microfluidic devices but in the presence of the reversible NCX blocker applied specifically to the axonal compartment where axotomy was performed (Figure 1B). We quantified spine density using repeated live imaging and found that in the presence of the NCX blocker, KB-R7943, spine loss was completely prevented (Figures 1B,C). Controls treated with vehicle had significantly fewer spines following axotomy (Figures 1B,C).

Sodium influx at the site of injury may trigger reversal of NCX needed for dendritic spine loss. Thus, we blocked sodium channels using tetrodotoxin (TTX) at the site of injury and quantified dendritic spine density changes. As expected, blocking sodium channels prevented a significant reduction in dendritic spine density (Figure 1D). Further, application of a potent activator of voltage gated sodium channels, veratridine, at distal axons and in the absence of injury led to a significant decrease in retrograde spine density 24 h post treatment. Together, these results show that sodium influx and reversal of NCXs at the site of injury trigger retrograde spine loss.

### Calcium Influx via Reversal of NCX Is Not Required to Induce Retrograde Hyper-Excitability Post-axotomy

**FIGURE 2:**
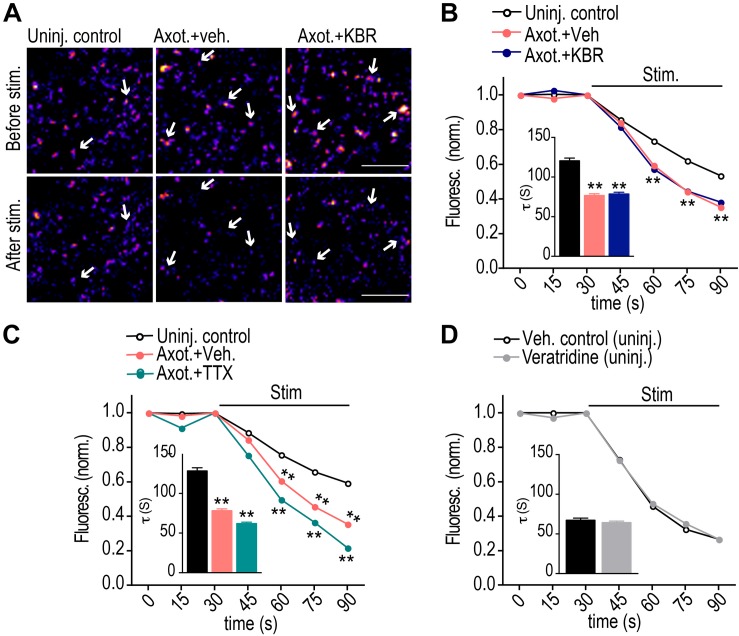
Calcium influx via reversal of NCX is not required to induce retrograde hyper-excitability post-axotomy. **(A)** Representative images of presynaptic terminals labeled with FM5-95 (FM puncta) before and after field stimulation within the somatodendritic compartment of microfluidic chambers. Color look up table ‘Fire’. Scale bars, 10 μm. **(B,C)** FM unloading curves at 48 h following application of KBR **(B)** or TTX **(C)** to axonal compartment for 1 h during axotomy. Uninjured control: *n* = 782 puncta; axotomy: *n* = 1037 puncta; Axot. KBR: *n* = 1012 puncta; Axot. TTX: 1415 puncta. **(D)** FM unloading curves at 48 h following 10 min application of veratridine to axonal compartment without injury. Control (DMSO): *n* = 711 puncta; veratridine: *n* = 1273 puncta. Two-way ANOVA, Bonferroni *post hoc* test. Inset in **B–D** shows FM decay time constant (τ) for puncta with τ < 360s (control: *n* = 654; Axot. Veh.: *n* = 977; Axot. KBR: *n* = 945 puncta; Axot. TTX: 1321 puncta). **(D)** control, *n* = 634; veratridine, *n* = 1177. Unpaired two-tailed *t*-test. 5–6 chambers for each condition over 3 independent experiments. ^∗∗^*p* < 0.001. Error bars, s.e.m.

We next tested whether retrograde hyper-excitability is triggered via reversal of NCXs. To examine retrograde hyper-excitability, we used FM dyes to quantify synaptic vesicle release dynamics as performed previously (Nagendran et al., 2017). This is an unbiased approach to measure synaptic vesicle release in response to field stimulation. FM dye is first loaded into recycling synaptic vesicles in response to KCl depolarization and then FM dye unloading is optically recorded in response to field stimulation to characterize the dynamics of synaptic vesicle release. Most, if not all, presynaptic terminals that formed onto injured neurons originate from uninjured neurons, as there was no detectable retrograde labeling of these presynaptic terminals (data not shown). Previously published data showed that synaptic vesicle release increases significantly 48 h post axotomy, analyzed via FM release curves and frequency of miniature excitatory postsynaptic currents (Nagendran et al., 2017). An advantage of this method is the ability to spatially observe release events localized to labeled and/or injured neurons. As a control we repeated these experiments and also tested whether blocking NCX reversal at the site of injury prevents the increase in hyper-excitability (Figures 2A,B). Surprisingly, we found that blocking NCX reversal at the site of injury did not prevent the increase in release rate due to axotomy. In fact, the KBR-treated cultures were indistinguishable from the vehicle controls. We performed a similar experiment using TTX (Figure 2C). Again, this did not prevent axotomy-induced hyper-excitability. In fact, the increase in release rate was exacerbated with the TTX treatment. We next tested whether veratridine in the absence of injury would cause hyper-excitability (Figure 2D). Consistent with our preceding data, the FM release curves were indistinguishable from vehicle controls. These results suggest that retrograde hyper-excitability involves a unique triggering mechanism independent from retrograde spine loss signaling following axotomy.

### Retrograde Hyper-Excitability Post-axotomy Requires Calcium Release From ER Intracellular Stores

**FIGURE 3:**
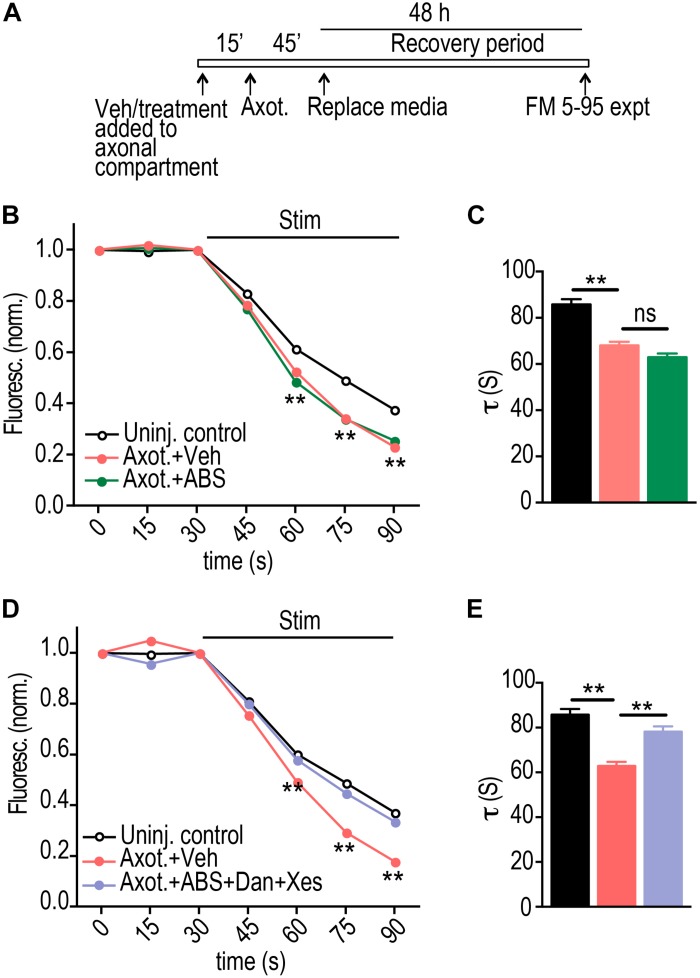
ER calcium channel activation at the site of injury is required for retrograde presynaptic hyper-excitability. **(A)** Experimental timeline for treatment and imaging within microfluidic chambers. **(B)** FM unloading curves at 48 h following application of vehicle or local activity blockade solution (ABS) to axons for 1 h during axotomy. Uninjured control: *n* = 1135 puncta; Axot. + Veh.: *n* = 1834 puncta; Axot. + ABS: *n* = 1590 puncta; 8 chambers per condition over 4 experiments. Two-way ANOVA, Bonferroni *post hoc* test. **(C)** FM decay time constant (*τ*) for puncta with *τ* < 360 s (Uninj. control: *n* = 1044; Axot. + Veh: *n* = 1686; Axot. + ABS: *n* = 1478). Unpaired two-tailed *t*-test. Each condition includes 8 chambers over 4 experiments. **(D)** FM unloading 48 h after application of vehicle or ABS supplemented with ryanodine receptor blocker (Dantrolene, 20 μM) and IP3 receptor blocker (Xestospongin C, 1 μM) to the axonal compartment. Uninjured control: *n* = 974 puncta; Axot. + Veh: *n* = 1239 puncta; Axot. ABS + Dan + Xes: 1124 puncta; 5 to 6 chambers per condition over 3 experiments. Two-way ANOVA, Bonferroni *post hoc* test. **(E)** FM decay time constant (*τ*) for puncta (Uninj. control: *n* = 892; Axot. + Veh: *n* = 1128; Axot. ABS + Dan + Xes: *n* = 1025). Unpaired two-tailed *t*-test. Each condition includes 5–6 chambers over 3 experiments. ^∗∗^*p* < 0.001. Error bars, s.e.m.

Calcium-mediated signaling is expected to be a critical factor in triggering retrograde hyper-excitability following injury. To further investigate the role of calcium, we applied low calcium and TTX solution within the axonal compartment at the time of injury. Surprisingly, this treatment did not alter the axotomy induced FM release kinetics compared with injured vehicle control (Figures 3A–C). Intracellular calcium stores may also play a critical role in axon-to-soma injury signaling, thus we next applied the low calcium/TTX solution together with blockers of ER calcium release from ryanodine (Dantrolene) and IP3 receptors (Xestospongin C). Blocking calcium release from ER normalized the synaptic vesicle release rate to uninjured control levels (Figures 3A,D,E).

**FIGURE 4:**
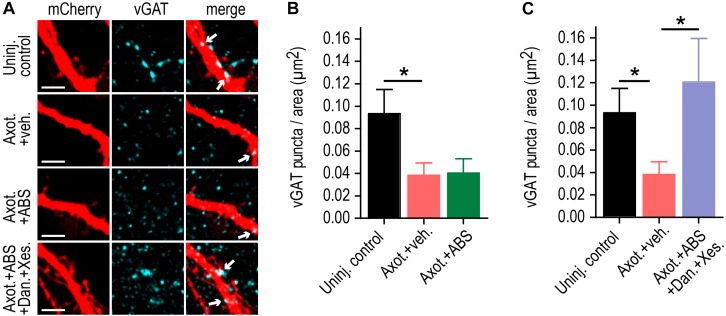
ER calcium channel activation at the site of injury is required for retrograde inhibitory terminal loss post-axotomy. **(A)** Representatrive mCherry labeled dendritic segments immunostained for the inhibitory presynaptic marker, vGAT, at 48 h following application of vehicle or local activity blockade solution (ABS) with or without Dantrolene and Xestospongin C to axons for 1 h during axotomy. Arrows in the merge image indicate the localization of vGAT puncta (cyan) on mcherry labeled dendrites (red). Scale bar, 5 μm. **(B,C)** Number of vGAT puncta per neuron area (uninjured control, axotomy or axotomy + ABS and axotomy + ABS + Dan + Xes) at 15-16 DIV. *n* = 6–8 neurons; 3 chambers per condition over 2 independent experiments. Error bars, SEM. ^∗^*p* < 0.05.

We previously found that the increase in FM release rate following injury coincided with loss of inhibitory terminals onto injured neurons. Loss of inhibition is a cause of hyper-excitability. As confirmation that low calcium/TTX did not substantially influence axotomy-induced disinhibition, we quantified the number of vGAT immunolabeled puncta colocalized with the dendritic arbor of labeled axotomized neurons in the presence of low calcium/TTX solution (Figures 4A,B). Application of this solution did not prevent the loss of inhibitory terminals. In contrast application of this solution with Dantrolene and Xestospongin C caused injured neurons to retain inhibitory terminals (Figure 4C).

**FIGURE 5:**
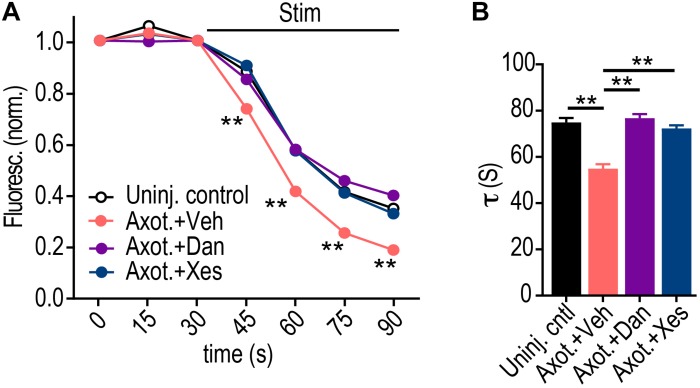
Dantrolene or Xestospongin C applied at the time and location of axotomy prevent retrograde enhancement of presynaptic release rate. **(A)** FM unloading curves at 48 h following application of vehicle or Dantrolene (20 μM) or Xestospongin C (1 μM) to axons for 1 h during axotomy. Uninjured control: *n* = 1076 puncta; Axot. + Veh: *n* = 893 puncta; Axot. + Dan: *n* = 1136 puncta; Axot. + Xes: *n* = 2234; 5 chambers per condition over two experiments. Two-way ANOVA, Bonferroni *post hoc* test. **(B)** FM decay time constant (*τ*) for puncta with *τ* < 360 s (Uninj. control: *n* = 1045; Axot. + Veh: *n* = 857; Axot. + Dan: *n* = 1058; Axot. + Xes: *n* = 2139). Unpaired two-tailed *t*-test. Each condition includes 5 chambers combined over 2 independent experiments. Error bars, SEM. ^∗∗^*p* < 0.005.

We next investigated the specificity of whether ryanodine or IP3 receptors may regulate retrograde presynaptic excitability changes without contributions from extracellular Ca^2+^. Ryanodine receptors, in particular, localize to hippocampal axons (Sharp et al., 1993). Using Dantrolene to block ryanodine receptors at the site of injury, we found that this alone was sufficient to block the retrograde presynaptic release rate enhancement (Figures 5A,B). We next tested the effect of axotomy when Xestospongin C is applied alone to the site of injury, and found that this also blocked the presynaptic release rate enhancement. Because ryanodine receptors are critical for Ca^2+^ induced Ca^2+^ release (CICR), it is possible that IP3 receptor activation may induce subsequent Ca^2+^ release via ryanodine receptors that are required for axon-to-soma signaling, thus explaining the dependence of both receptors on the retrograde enhancement in release rate (see section “Discussion”). Together, these data show that ER provides a critical source of Ca^2+^ needed to propagate the *trans*-synaptic change of altering synaptic vesicle release.

### ER-Dependent Changes Following Axotomy

**FIGURE 6:**
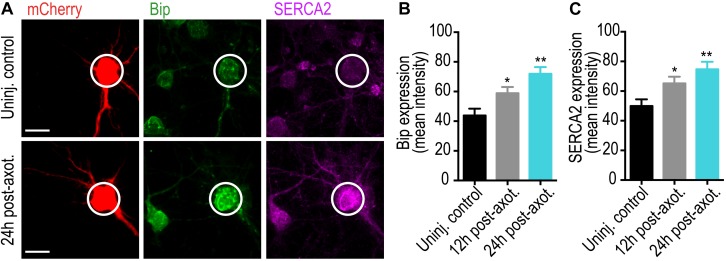
Axotomy induces somatic ER changes within our microfluidic model. **(A)** Representative images of retrograde labeled mCherry neurons co-immunostained with the Ca^2+^ binding protein and ER stress marker, GRP78/BiP, and the ER Ca^2+^ pump, SERCA2. Scale bar, 10 μm. White circles highlight the soma of axotomized and uninjured control neurons. Quantification of somatic BiP **(B)** and SERCA2 **(C)** expression at 12 and 24 h post-axotomy are indicative of changes in somatic Ca^2+^ homeostasis. *N* = 18 neurons; 2 chambers per condition. Error bars, SEM. ^∗^*p* < 0.05, ^∗∗^*p* < 0.005.

Our data suggests that ER plays a critical role in *trans*-synaptic injury signaling following axotomy. To determine whether somatodendritic ER changes following axotomy, we measured the changes in the Ca^2+^ binding protein and ER stress marker, BiP, in the somata of injured neurons using immunolabeling (Figures 6A,B). Significant BiP accumulation occurred within the soma at 12 and 24 h post-axotomy. We also found significant upregulation of the ER Ca^2+^ pump, SERCA2, within the somatodendritic compartment following axotomy (Figures 6A,C). These data support ER-dependent changes in Ca^2+^ homeostasis in somata following axotomy.

## Materials and Methods

### Microfluidic Chambers

Poly (dimethylsiloxane) (PDMS) was molded onto a SU-8 master with 900 μm long, 3–4 μm tall and 7.5–8 μm wide microgrooves as previously described (Taylor et al., 2003, 2005). Chambers were sterilized in 70% ethanol and placed onto 500–550 kDa Poly-D-Lysine (BD Biosciences) coated sterile German glass coverslips.

### Hippocampal Cultures

Animal procedures were approved by the University of North Carolina at Chapel Hill Institutional Animal Care and Use Committee (IACUC). Sprague Dawley rat embryos (E18-E19) were used to prepare dissociated hippocampal cultures as previously described (Nagendran et al., 2017). Hippocampal cells were dissociated in neuron culture media i.e., neurobasal media (Invitrogen) supplemented with 1 X B27 (Invitrogen), 1 X Antibiotic-antimycotic (Invitrogen), and 1 X Glutamax (Invitrogen). Approximately ∼90,000 cells were plated into the somatodendritic compartment of the chamber. After 5–7 days of culture in microfluidic chambers, axons extended into the adjacent axonal compartment.

### Dendritic Spine and Retrograde Neuron Labeling

Dendritic spines of injured neurons were identified using G-deleted Rabies-mCherry or eGFP virus (Nagendran et al., 2017). Neurons were infected between 11 and 13 days *in vitro* (Wickersham et al., 2007) (Salk Institute; 1 × 10^5^ viral units) as previously described (Nagendran et al., 2017). G-deleted Rabies-mCherry or eGFP virus diluted in 200 μl neuron culture media was added to the axonal compartment of each chamber. After 2 h incubation with virus at 37°C remove virus containing media from the axonal compartment. Saved conditioned media was added back to the axonal compartments following two washes with fresh culture media. Chambers were maintained in 37°C incubator for ∼48 h until fluorescence expression was visible. Axotomy was performed between 13 and 15 days *in vitro* (DIV) as previously described (Nagendran et al., 2017).

### Immunocytochemistry

Neuronal cultures were fixed with 4% PFA and permeabilized in 0.25% Triton X-100. Coverslips were blocked in 10% normal goat serum for 15 min each and incubated with anti-vGLUT1 (1:100; NeuroMab, clone N28/9, #75-066), anti-vGAT (1:1000; Synaptic Systems #131 003), anti-SERCA (1:500; Abcam # ab2817), and anti-GRP78 Bip (1:200; Abcam # ab21685) primary antibodies in 1% blocking solution for overnight at 4°C. Coverslips were then incubated with goat anti-rabbit or goat anti-mouse secondary antibodies conjugated to Alexa-fluorophores (1:500; Invitrogen) for 1 h at RT.

### FM Dye Experiments and Analysis

Recycling synaptic vesicles of 48 h (15 DIV) post-axotomy hippocampal cultures were loaded with lipophilic dye N-(3-trimethylammoniumpropyl)-4-(6-(4-(diethylamino) phenyl) hexatrienyl)pyridinium dibromide (FM 5–95; Invitrogen) using KCl mediated depolarization as described previously (Taylor et al., 2013). For FM unloading, microfluidic chambers were stimulated using extracellular electrodes by placing a positive and negative electrode in each well of the somatodendritic compartment. Electrical stimulation was provided by an AD Instruments 2 Channel Stimulus Generator (STG4002) in current mode with an asymmetric waveform (−480 μA for 1 ms and + 1600 μA for 0.3 ms) for ∼1 min at 20 hz for 600 pulses. The FM 5–95 imaging was performed using a spinning disk confocal imaging system as previously described in Taylor et al. (2013). Z-stacks (31 slices) were captured every 15 s during the baseline (1 min), stimulation (1 min), and after stimulation (2 min) periods. At least 3 baseline images were acquired before electrical stimulation. Sum projected confocal z-stack were converted to 8-bit images and registered using TurboReg, an Image J plugin. We background subtracted the image stack using the image 3 min after stimulation began as described in Nagendran et al. (2017). Briefly, image stacks were thresholded to a pixel value of 15. FM puncta between 0.4 to 10 μm^2^ were analyzed. We measured the intensity of each punctum in the whole field throughout all time series. We normalized fluorescence intensity of each puncta to the frame before stimulation. Puncta with >5% unloading after 1 min were used in the analysis as unloaded puncta. Time constants were estimated by curve fitting unloading kinetics to a single exponential decay function (Taylor et al., 2013). Curve fitting was done in MATLAB and FM puncta with time constants longer than 3 min were excluded from the analysis.

### Drug Treatments

KB-R7943 (Tocris Bioscience # 1244) was suspended in DMSO and applied to the axonal compartment at a final concentration of 10 μM for 1 h during axotomy (including 15 min pre-incubation before axotomy). Tetrodotoxin citrate (TTX; Tocris Bioscience #1078) was suspended in HBS and applied to the axonal compartment at a final concentration of 1 μM for 1 h during axotomy (beginning 15 min prior to axotomy). Veratridine (Tocris Bioscience #2918) was suspended in DMSO and applied to the axonal compartment at a final concentration of 10 μM for 10 min in the absence of axotomy/injury. Local ABS, which includes low-Ca^2+^, high-Mg^2+^, and TTX (0.5 mM CaCl_2_, 10 mM MgCl_2_, 1 μM TTX) was applied solely to the axonal compartment for 1 h during axotomy (15 min prior and 45 min after axotomy). Dantrolene (Tocris Bioscience #0507) and (-)-Xestospongin C (Tocris Bioscience #1280), stock concentrations prepared in DMSO, were diluted in HBS or ABS solution and added to axonal compartment at a final concentration of 20 and 1 μM respectively for 1 h during axotomy (with 15 min pre-treatment and 45 min treatment post-axotomy). DMSO or HBS was used as vehicles. Media stored from the axonal compartment prior to treatment was added back to the axonal compartment after treatment and washes with pre-warmed fresh neuron culture media.

### Microscopy and Image Analysis

FM and fixed imaging was performed using CSU-X1 (Yokogawa) spinning disk confocal imaging unit configured for an Olympus IX81 microscope (Andor Revolution XD). Excitation for the spinning disk confocal imaging system was provided by 405 nm, 488 nm, 561 nm, and/or 640 nm lasers. The following bandpass emission filters (BrightLine, Semrock) were used for the spinning disk: 447/60 nm (TRF447-060), 525/30 nm (TRF525-030), 607/36 nm (TR-F607-036), and 685/40 nm (TR-F685-040). Zeiss LSM 780 (63 × 1.4 NA or 40 × 1.4 NA oil immersion objective) or the spinning disk system above (60 × 1.3 NA silicon oil immersion objective) was used to capture high-resolution images of mCherry or eGFP labeled live neurons as previously described (Nagendran et al., 2017).

For FM imaging, the spinning disk confocal imaging system was used with excitation at 561 nm and the 685/40 nm emission filter. We used 2 × 2 binning to reduce the laser intensity and acquisition time for each frame; each z-stack was obtained in ∼5 s. For dendritic spine analysis, before and 24 h post-axotomy confocal z-stack images of live G-deleted Rabies-mCherry or eGFP virus labeled neurons were captured to create montages of neurons extending axons into the axonal compartment. Fluorescent protein labels the entire neuron including small protrusions like dendritic spines. Calibrated z-stack montages were analyzed for all dendrite and spine parameters. Primary dendrites were traced using the semiautomatic neurite tracing tool, Neuron J (Meijering et al., 2004). Dendritic spines were quantified based on their size and shape. The number of spines on all primary dendrites of each neuron was manually counted and spine density was calculated for 10 μm length of dendrite as [(# of spines/dendrite length) × 10] (Nagendran et al., 2017).

### Statistical Analysis

Statistical analyses were performed using GraphPad Prism 6. Spine density before and after treatment or injury was analyzed using paired two-tailed *t*-test. FM unloading curves were analyzed by two-way ANOVA, using a Bonferroni *post hoc* test. FM decay time constants were analyzed using either unpaired two-tailed *t*-test. vGat puncta per area were analyzed by unpaired two-tailed *t*-test. For sample size, *p*-value, and statistical test, refer to the figure legends.

## Discussion

Hyper-excitability following acquired brain injury leads to long-term effects, such as persistent seizures, chronic pain, and spasticity. Intrinsic injury signaling within damaged neurons likely plays a key role in induced hyper-excitability.

The main conclusion of our results is that axonal ER signaling plays a critical role in regulating axotomy-induced retrograde presynaptic release in hippocampal neurons. Using our *in vitro* microfluidic model, our data show that while NCXs at the site of injury mediate retrograde dendritic spine loss, they do not mediate the delayed retrograde changes in presynaptic release rate. Rather than influx of extracellular calcium into the cytosol, release from internal ER stores within the axon at the site of injury appears to be a critical signaling component that triggers changes in retrograde presynaptic neurotransmitter release rate.

Dantrolene or Xestospongin C applied at the time and location of axotomy, both prevented the injury-induced retrograde release changes. Both receptors are localized to hippocampal neurons. RyRs are predominant in hippocampal CA3 and DG with less expression in CA1 (Sharp et al., 1993). IP3Rs show the opposite trend, though there is evidence of immunoreactivity of both receptors in the same neuron. Interestingly, RyRs are more prominently seen in hippocampal axons than IP3Rs (Sharp et al., 1993).

RyRs play a critical role in injury-dependent neuronal signaling. Inhibiting RyRs with Dantrolene reduced neuronal injury in an ischemic gerbil model (Wei and Perry, 1996). RyR are also necessary for CICR, suggesting the involvement of this mechanism in presynaptic release changes following axotomy (Verkhratsky, 2005). Ca^2+^ release from IP3Rs may induce RyR-mediated Ca^2+^ release. Xestospongin C blocks SERCA pumps in addition to IP3R, preventing Ca^2+^ uptake into the ER (Smet et al., 1999; Castonguay and Robitaille, 2002; Solovyova et al., 2002). Thus, it is also possible that the non-specific effect of this drug may alter ER calcium availability via mechanisms not involving IP3Rs and may explain why the retrograde release changes were prevented with local application of this drug. Nonetheless, a major conclusion of this data is the dependence of Ca^2+^ release from ER, and not extracellular Ca^2+^ influx, for the axotomy-induced enhancement in retrograde presynaptic release rate.

Our previously published data support that both axotomy-induced dendritic spine loss and presynaptic release changes are mediated by rapid transcription (Nagendran et al., 2017). Our data show that ER changes in Ca^2+^ buffering and sequestration are observed in the soma following axotomy (Figure 6), supporting axon-to-soma ER-dependent Ca^2+^ signaling. In peripheral neurons, locally initiated calcium waves can propagate to the nucleus to induce a transcriptional response (Cho et al., 2013). Other studies suggest that local influx of calcium may be a priming effect for retrograde microtubule-based transport of signaling complexes required to initiate transcription (Rishal and Fainzilber, 2014). We previously identified netrin-1, a known synaptogenic cue, as downregulated within the somatodendritic compartment both *in vivo* and *in vitro* following axon damage (Nagendran et al., 2017). Further application of exogenous netrin-1 after axotomy normalized both dendritic spine density and inhibitory terminals, suggesting netrin-1 signaling may regulate axotomy-induced synapse loss. Interestingly, ER stress activates IRE1, a sensor that activates UPR, to degrade netrin-1 mRNA (Binet et al., 2013).

Critical involvement in ER signaling is not only implicated in acute neural injuries, but also in multiple neurological disorders (Ozcan and Tabas, 2012), including Alzheimer’s disease, Parkinson’s disease, multiple sclerosis, amyotrophic lateral sclerosis, and prion diseases. In these diseases, axon damage is a first site of pathology, suggesting signaling from the axon may be a key early event and clear target for future therapeutics.

## Data Availability STATEMENT

The datasets generated for this study are available on request to the corresponding author.

## ETHICS STATEMENT

The animal study was reviewed and approved by The University of North Carolina at Chapel Hill Institutional Animal Care and Use Committee (IACUC).

## Author Contributions

TN designed the experiments, acquired the data, analyzed the data, and wrote the manuscript. AT designed the experiments and wrote the manuscript.

## Conflict of Interest

AT is an inventor of the multi-compartment microfluidic device (US 7419822 B2, EPO 1581612, and EPO 2719756), and is Chief Scientist and a Member of Xona Microfluidics, LLC.

The remaining author declares that the research was conducted in the absence of any commercial or financial relationships that could be construed as a potential conflict of interest.
